# Modification of a Volume-Overload Heart Failure Model to Track Myocardial Remodeling and Device-Related Reverse Remodeling

**DOI:** 10.5402/2011/831062

**Published:** 2011-07-06

**Authors:** Egemen Tuzun, Roger Bick, Cihan Kadipasaoglu, Jeffrey L. Conger, Brian J. Poindexter, Igor D. Gregoric, O. H. Frazier, Jeffrey A. Towbin, Branislav Radovancevic

**Affiliations:** ^1^Cardiovascular Surgical Research Laboratories, Texas Heart Institute, Houston, TX 77225, USA; ^2^Texas A&M Institute for Preclinical Studies, 800 Raymond Stotzer Pkwy, Ste 2060, College Station, TX 77843-4478, USA; ^3^Department of Pathology, University of Texas Health Science Center, Houston, TX 77030-1501, USA; ^4^Department of Pediatric Cardiology, Baylor College of Medicine, Houston, TX 77030, USA

## Abstract

*Purpose*. To provide an ovine model of ventricular remodeling and reverse remodeling by creating congestive heart failure (CHF) and then treating it by implanting a left ventricular assist device (LVAD). *Methods*. We induced volume-overload heart failure in 2 sheep; 20 weeks later, we implanted an LVAD and assessed recovery 11 weeks thereafter. We examined changes in histologic and hemodynamic data and levels of cellular markers of CHF. *Results*. After CHF induction, we found increases in LV end-diastolic pressure, LV systolic and diastolic dimensions, wall thickness, left atrial diameter, and atrial natriuretic protein (ANP) and endothelin-1 (ET-1) levels; *β*-adrenergic receptor (BAR) and dystrophin expression decreased markedly. Biopsies confirmed LV remodeling. After LVAD support, LV systolic and diastolic dimensions, wall thickness, and mass, and ANP and ET-1 levels decreased. Histopathologic and hemodynamic markers improved, and BAR and dystrophin expression normalized. *Conclusions*. We describe a successful sheep model for ventricular and reverse remodeling.

## 1. Introduction

Congestive heart failure (CHF) is characterized by an initial cardiac insult, followed by a compensatory response that results in ventricular remodeling. At the cellular level, CHF involves myocyte hypertrophy, cell death, and replacement fibrosis. At the subcellular level, the disease causes loss of myofilaments, alterations in cytoskeletal proteins, and adrenergic desensitization. 

For the past 2 decades, the diagnosis of heart failure has relied on noninvasive methods (physical examination, chest roentgenography, electrocardiography, and echocardiography) and invasive tests [1]. Various biomarkers have also been used to diagnose heart failure with marginal clinical utility. In patients with ischemic heart disease and myocardial infarction, the following biomarkers have shown varying levels of sensitivity in detecting heart failure: lactic dehydrogenase, creatine kinase myocardial band (CKMB), troponins T and I, C-reactive protein, norepinephrine, endothelin-1 (ET-1), interleukin-6, tumor necrosis factor-*α* (TNF-*α*), atrial natriuretic protein (ANP), B-type natriuretic peptide, beta-adrenergic receptors (BARs), and angiotensin II [2–12]. Another cellular marker of heart failure is dystrophin, a protein that connects the extracellular matrix with the sarcomere via actin. Dystrophin abnormalities have been associated with heart failure caused by genetic susceptibility and viral infection [13]. Treatment of CHF with left ventricular assist devices (LVADs) can reverse the ventricular remodeling associated with heart failure, and this reversal is correlated with normalization of cellular markers in myocytes [14, 15]. The present pilot study was undertaken to provide an ovine experimental model for creating CHF, then reversing this condition by implanting an LVAD. We assessed the changes in immunohistochemical, histopathologic, and hemodynamic parameters to verify the occurrence and extent of the remodeling events in heart failure development (remodeling) and subsequent LVAD-assisted recovery (reverse remodeling).

## 2. Materials and Methods

### 2.1. Animal Model

Two male Rambouillet sheep (54 ± 4 kg) were used in this study. The animals received humane care in compliance with the *Principles of Laboratory Animal Care *(National Society of Medical Research) and the *Guide for the Care and Use of Laboratory Animals* (National Institutes of Health Publication no. 85-23, revised 1996). Our Institutional Animal Care and Use Committee approved the protocol in advance.

To accurately mimic the clinical scenario of ventricular remodeling in heart failure, we created a volume-overload heart-failure model by destroying the chordae tendineae of each sheep's mitral valve. After 20 weeks, we implanted a Levitronix CentriMag (Levitronix LLC, Waltham, Mass) (1st case) and a HeartWare LVAD (HeartWare International, Inc., Framingham, Massachusetts) (2nd case) to support the animals. After 11 weeks of LVAD support, the sheep were reexamined for evidence of myocardial recovery.

### 2.2. Operative Procedures

#### 2.2.1. Anesthesia

We followed a standard institutional anesthesia protocol that has previously been described in detail [16]. Each sheep was premedicated with glycopyrrolate (0.02 mg/kg) and xylazine (0.2–0.7 mg/kg); both drugs were administered intramuscularly. Intravenous ketamine (10–20 mg/kg) was administered to induce anesthesia. General anesthesia was maintained with isoflurane (1–3%) in oxygen (40–100%).

#### 2.2.2. 1st Operation: Creation of Mitral Regurgitation (Baseline)

**Figure 1:**
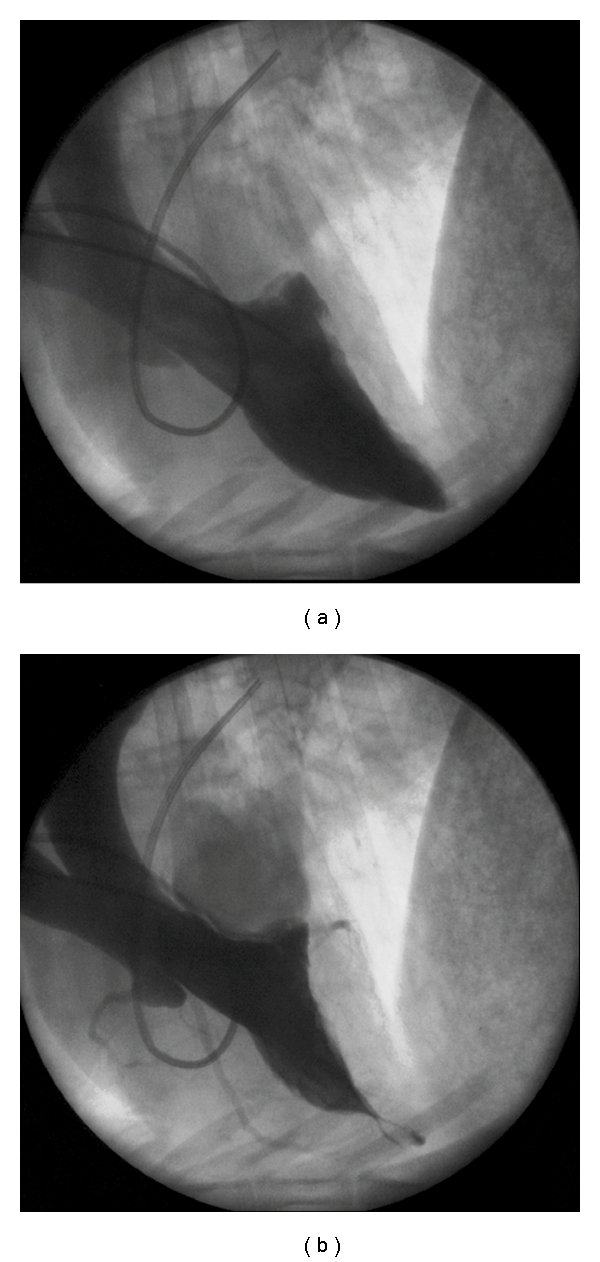
(a) Normal ventriculogram obtained before creation of mitral regurgitation (MR). (b) After chordal disruption, the ventriculogram shows 2nd- to 3rd-degree MR.

Transthoracic echocardiography was performed to obtain baseline measurements of the left and right ventricular dimensions, left ventricular mass, ventricular wall thickness, and valve function. The baseline left ventricular end-diastolic pressure (LVEDP) was measured with a micromanometer-tip pigtail catheter (Millar Mikro-Tip Catheter; Millar Instruments, Houston, Tex) inserted via the left carotid artery. After baseline hemodynamic measurements were made, baseline endomyocardial biopsy specimens were taken from the left ventricle via a transaortic route. With the sheep in stable condition, a baseline left ventriculogram was performed to assess mitral valve function. The pigtail catheter was removed, and a reusable biopsy and retrieval forceps (Cook Urological Inc., Spencer, Idaho) were introduced into the left ventricle. Under fluoroscopic guidance, this forceps was used to grasp and cut 1 or more chordae tendineae upon forceful withdrawal, creating severe MR (3+ to 4+ Seller's grade [17]) (Figures 1(a) and 1(b)). This condition was defined by a high degree of opacification of the left atrium 2 beats after left ventricular contrast injection. After creation of the MR, the left carotid artery was repaired, and the sheep was allowed to recover. This procedure has previously been described elsewhere [18]. During the follow-up period, the sheep underwent echocardiography every 2 weeks to detect volume-overload heart failure.

#### 2.2.3. 2nd Operation: Implantation of an LVAD (20th Week)

**Levitronix CentriMag LVAD**

The Levitronix CentriMag LVAD is a sterile, single-use, disposable, and centrifugal pump. The pump's 32-mL priming volume minimizes the wetted surface area and helps to limit the need for intravenous fluids during surgery. The pump inlet is on the rotational axis of the rotor; the pump outlet is perpendicular to the inlet and tangential to the outer diameter. Both the inlet and outlet ports are standardized 3/8-inch barbed connectors for easy application to standard medical-grade 3/8-inch tubing. The blood pump design is based on magnetic-levitation motor-bearing technology that allows pumping without mechanical bearings and seals. The pump's rotor “floats” in a rotating magnetic field without mechanical contact, and an external compact digital signal processor system allows precise regulation of the rotor's speed. The pump is designed to provide flow in the range of 0.5 to 9.9 L/min.

The drive console is a microprocessor-based system that includes user interface keys which provide convenient management points for pump operation; a 4-line alphanumeric display for showing the system status; alert and alarm information; 27-segment digital displays that show the pump speed and blood flow rate.

**HeartWare LVAD**

The HeartWare LVAD is described in detail elsewhere [19]. Briefly, the HeartWare LVAD is a small, wearless, and centrifugal blood pump. It has a displaced volume of just 45 cc, weighs 145 g, and can deliver flows of up to 10 L/min.

**Implantation Technique**

For implantation of the CentriMag LVAD, anesthesia was induced routinely, and hemodynamic, echocardiographic, and fluoroscopic measurements were repeated as were the endomyocardial biopsies. A left thoracotomy was then performed in the 5th intercostal space, and the left ventricular apex and descending aorta were exposed for inflow and outflow cannula placement, respectively. After 2 pledgeted, 2-0 braided polyester pursestring sutures were placed on the left ventricular apex and descending aorta, 32F inflow and 20F outflow cannulas were inserted into these 2 structures, respectively. The procedures were done on the beating heart without cardiopulmonary bypass. After the cannulas were tunneled outside the chest wall, they were connected to a centrifugal CentriMag extracorporeal LVAD, and flow was initiated at 4 L/min. Pump flow was measured throughout the study with a 10-mm flow probe (Transonic Systems Inc., Ithaca, New York) on the outflow graft cannula, and unloading of the left ventricle was adjusted according to that chamber's size. Device flow was maintained between 5 and 7 L/min. The LVAD was secured to each sheep's body with a girth strap. Postoperatively, the animals were treated the same as after the initial operation. Adjunctive esmolol infusion and diuretics were used to control tachycardia and peripheral vascular resistance/urine output, respectively. The animals were given warfarin to keep the international normalized ratio between 2.5 and 3.5.

The same anesthesia and postoperative protocols were used for HeartWare implantation, which has been detailed elsewhere [19].

#### 2.2.4. 3rd Operation: Hemodynamic, Echocardiographic, and Fluoroscopic Studies after 11 Weeks of LVAD Support (31st Week)

Eleven weeks after placing the LVAD, we remeasured the hemodynamic, echocardiographic, and fluoroscopic variables and repeated the endomyocardial biopsies with the sheep under general anesthesia.

### 2.3. Echocardiographic Assessment

Echocardiographic assessment was performed according to the guidelines of the American Society of Echocardiography [20] by an echocardiologist using a Sonos 2000 ultrasound system (Hewlett-Packard Co., Palo Alto, Calif) equipped with a 2.5-MHz phased-array transducer.

### 2.4. Histopathologic Evaluation

Endomyocardial biopsies were performed before MR creation (baseline), during the 2nd operation (week 20), and during the 3rd operation (week 31) and were evaluated with transmission electron microscopy. Macroscopic postexplant analyses of the pump were also done after termination of the study.

### 2.5. Immunohistochemical Staining

**β**-adrenergic receptor, ANP, and ET-1 levels were selected as markers of heart failure and were fluorescently labeled. Fluorescence was quantified as pixel densities of specific colors in split color images, using Corel Draw (Corel Corporation, Ottawa, ON, Canada) and Sigma Scan Pro software (Aspire Software International, Leesburg, Va). 

### 2.6. Quantification of Dystrophin in Ventricular Biopsy Specimens

Expression of dystrophin was quantified by Western blotting of whole tissue lysates prepared from biopsy tissues. Consequently, confocal immunofluorescence was used to obtain robust quantitative information about the number of immunoreactive signals at discrete sites within the cell.

## 3. Results

### 3.1. Induction (Remodeling Phase) and Treatment (Reverse Remodeling Phase) of Heart Failure

In both cases, comparison of hemodynamic, fluoroscopic, echocardiographic, histopathologic, and immunohistochemical data recorded (1) during the 1st operation and (2) immediately before pump implantation confirmed the effectiveness of the heart failure induction protocol. 

After the 2nd operation, both sheep recovered from anesthesia without complications and were extubated within the 1st postoperative hour. Eleven weeks after LVAD implantation, they reached the scheduled endpoint without experiencing anorexia, infection, or a neurologic disorder.

### 3.2. Device Examination and Animal Necropsy

The explanted pumps' interior, inflow cannula, and outflow cannula were completely free of thrombus. There was no evidence of ischemia or infarction in any of the peripheral end organs (brain, liver, spleen, and kidneys).

### 3.3. Hemodynamic Data

The mean LVEDP, which was 8 ± 2 mmHg at baseline, increased to 16 ± 2 mmHg immediately after chordal disruption and to 27 ± 4 mmHg during the 2nd operation. After 11 weeks of LVAD support, the mean LVEDP measured 14 ± 2 mmHg.

### 3.4. Fluoroscopic Data

After the mitral chordae tendineae were disrupted, left ventriculography confirmed the presence of 2nd- to 3rd-degree MR (Figures 1(a) and 1(b)). During the 2nd operation, repeat ventriculography showed 4th-degree MR and a dilated left atrium. After 11 weeks of LVAD support, ventriculography revealed 4th-degree MR and a dilated left atrium at a pump flow of 5 L/min in both sheep.

### 3.5. Echocardiographic Data

Table 1 shows the mean echocardiographic results obtained during the 1st operation (after mitral chordal disruption), 2nd operation (LVAD implantation), and 3rd operation (after 11 weeks of LVAD support). The echocardiographic data demonstrate that volume-overload heart failure was successfully created in both animals, with a resulting increase in the left ventricular diastolic dimension (LVDD), left ventricular systolic dimension (LVSD), and left ventricular mass. After LVAD support, the echocardiographic measurements improved, with a substantial decrease in the LVDD, LVSD, and left ventricular mass.

### 3.6. Histopathologic Data

**Figure 2:**
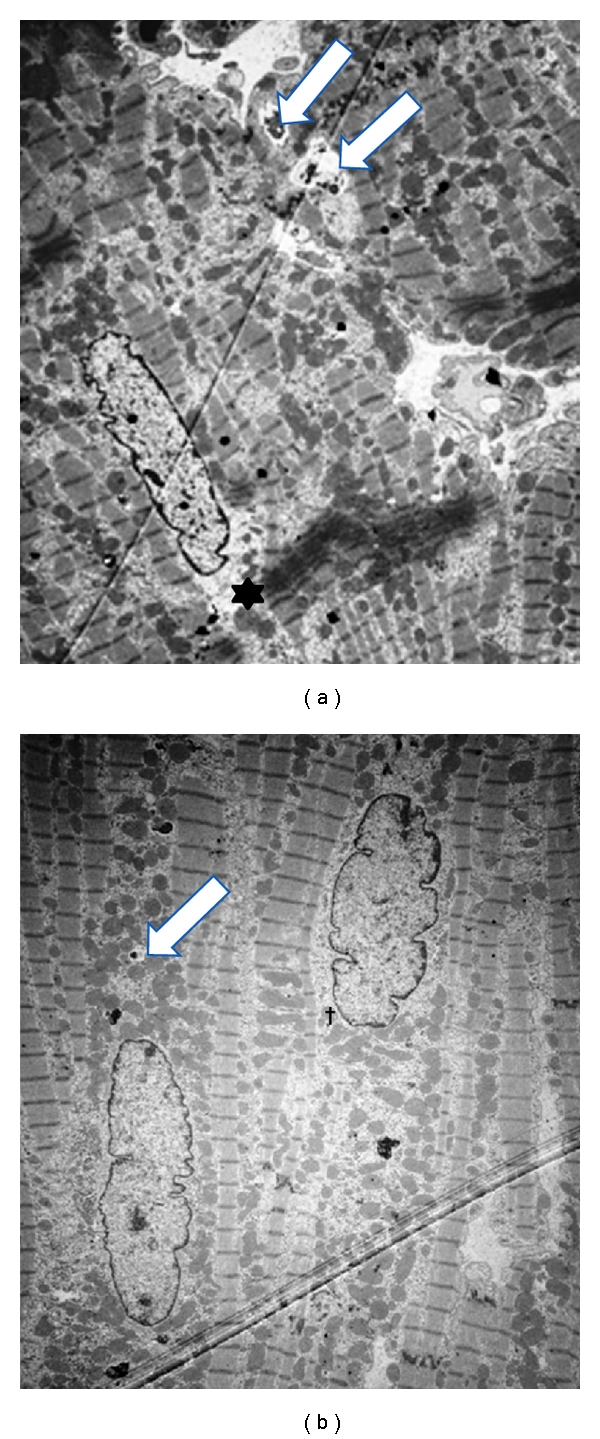
(a) Biopsy specimen obtained at left ventricular assist device (LVAD) implantation, showing a loss of myofilaments in the perinuclear region (asterisk) and the presence of cytoplasmic vacuoles (arrows). (b) Biopsy specimen obtained after 11 weeks of LVAD unloading, showing a reduced loss of myofilaments, with fewer and smaller cytoplasmic vacuoles (arrow) and more and larger mitochondria (dagger). Magnification ×2500; scale bar 2 *μ*M.

Transmission electron microscopy of the myocardial biopsy specimens obtained during LVAD placement (2nd operation) showed a 58 ± 5% loss of myofilaments in the perinuclear region; the presence of cytoplasmic vacuoles >1.5 *μ*m in diameter decreased by 33  ±  4% compared to baseline biopsy findings (Figure 2(a)).

After 11 weeks of LVAD unloading, the percentage of cells with moderate to severe myofilament loss decreased from 58 ± 5% (at LVAD implantation) to 18 ± 3%. The percentage of cells with cytoplasmic vacuoles >1.5 *μ*m in diameter decreased from 33 ± 4% to 5 ± 2%. Cardiac myocytes showed an increase in the number and size of their mitochondria (Figure 2(b)).

### 3.7. Immunohistochemical Data

**Figure 3:**
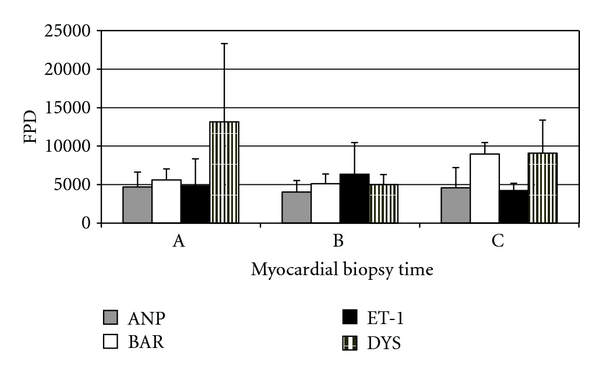
Identification of markers in heart failure and recovery. The results are expressed as the mean and standard error for each time point: before induction of heart failure (a), after induction of heart failure (b), and after 11 weeks of LVAD support (c). ANP: atrial natriuretic protein; BAR: *β*-adrenergic receptors; ET-1: endothelin-1; DYS: dystrophin; FPD: fluorescence pixel density.

Compared to baseline values, there was a marked increase in the fluorescence pixel density for ANP and ET-1 along with a reduction in BAR and dystrophin density (Figure 3), which is evidence of volume-overload heart failure.

After 11 weeks of LVAD support, biopsy samples showed a marked decrease in fluorescence pixel density for ANP and ET-1, along with an increase in BAR and dystrophin density (Figure 3) which may be considered a sign of myocardial reverse remodeling.

## 4. Discussion

In our pilot study, the left ventricular diastolic and systolic dimensions increased with heart failure and decreased after LVAD support, although baseline levels were not regained after 11 weeks of LVAD support. The cardiac index and left ventricular mass were elevated during heart failure and returned to near baseline levels after 11 weeks of LVAD support. Together, these results indicated the successful development of heart failure in sheep after chordal disruption, followed by recovery after LVAD support, as determined by physical measurements and hemodynamic data.

We used an adult sheep model, primarily because of the ease with which heart failure can be induced and subsequent LVAD therapy can be applied in this model. Other fundamental advantages of the sheep are the animal's size (which allows placement of a human-sized LVAD), minimal growth rate, ease of management, and hemodynamic similarities to humans. Moreover, the ability to perform sequential biopsies allowed us to monitor the progress of heart failure and to correlate the extent of remodeling and its reversal with ANP, ET-1, BAR, and dystrophin levels. Because our volume-overload heart failure ovine model was not a previously established one, biopsies to detect heart failure creation (remodeling) and postimplant recovery timing (reverse remodeling) were scheduled and performed according to the guiding results of the echocardiographic follow-up data. The echocardiographic findings of volume-overload heart failure (such as ejection fraction, left atrial diameter, left ventricular end-systolic and end-diastolic dimensions, ventricular mass and thickness, etc.) and LVAD recovery correlated well with the animal's clinical picture and hemodynamics, so we also scheduled LVAD implantation and support times according to these echocardiographic results. 

The changes in dystrophin expression in our pilot study were consistent with the development of heart failure and subsequent recovery after LVAD placement in humans [14]. Because dystrophin has been demonstrated to play a key role in linking the actin cytoskeletal networks and the sarcolemmal dystrophin-associated protein complex, the lack of dystrophin is associated with altered structure and mechanical adherence of costameres to the underlying cytoskeletal actin network, resulting in cardiomyopathy [15]. Therefore, the possible fragility (disruption) of the dystrophin-actin bonds may be important for tracking early myocardial structural changes in remodeling and reverse remodeling and for developing a more precise animal model of heart failure to test the efficacy of short- or long-term LVAD support. 

One of our study's limitations was the lack of plasma profiles of dystrophin levels, which may be used as a predictive/clinical biomarker for left ventricular remodeling related to LVAD use. Such plasma profiles will be a main focus of our future studies involving a larger number of animals. Moreover, the location of the outflow-graft anastomosis may be considered another study limitation, as it may not be the anastomotic site currently preferred for most fully implantable LVAD outflow grafts.

## 5. Conclusion

In this pilot experimental study, we have demonstrated an experimental animal model in which we created CHF in 2 adult sheep and subsequently reversed this condition by implanting 2 different centrifugal LVADs (1 device in each animal). The degree of remodeling was indicated by the disruption of dystrophin expression, as well as hemodynamic, echocardiographic, histologic, molecular, and clinical signs of heart failure. The reversal of heart failure (myocardial recovery), by means of mechanical circulatory assistance, was indicated by the normalization of dystrophin expression and integrity. We believe that this technique for creating and subsequently treating CHF with LVAD therapy may be useful in future studies investigating the diagnosis and prevention of CHF.

## Figures and Tables

**Table 1:** Mean echocardiographic measurements obtained in each individual sheep during the 1st operation (after mitral chordal disruption), 2nd operation (left ventricular assist device (LVAD) implantation), and 3rd operation (after 11 weeks of LVAD support).

Time	LVDD (mm)	LVSD (mm)	IVS (mm)	PW (mm)	RVDD (mm)	LA (mm)	EF (%)	LV mass (g)
*Sheep 1*								
1st operation (baseline)	32	22	7	7	19	32	55	201
2nd operation (wk 20)	59	44	11	9	26	48	44	570
3rd operation (wk 31)	41	26	11	10	29	48	56	210
*Sheep 2*								
1st operation (baseline)	36	26	9	9	25	36	65	233
2nd operation (wk 20)	68	50	13	11	30	56	50	610
3rd operation (wk 31)	45	30	13	12	35	56	64	236

EF: ejection fraction; IVS: interventricular septal thickness; LA: left atrial dimension; LV: left ventricular; LVDD: left ventricular diastolic dimension; LVSD: left ventricular systolic dimension; PW: posterior wall thickness; RVDD: right ventricular diastolic dimension.
